# Glyphosate as an Emerging Environmental Pollutant and Its Effects on Breast Cancer Cell Proliferation: A Systematic Literature Review of Preclinical Evidence

**DOI:** 10.3390/toxics14010026

**Published:** 2025-12-26

**Authors:** Mario A. Alcalá-Pérez, Gustavo A. Hernández-Fuentes, Idalia Garza-Veloz, Uriel Diaz-Llerenas, Margarita L. Martinez-Fierro, José Guzmán-Esquivel, Fabian Rojas-Larios, Ángel A. Ramos-Organillo, Kayim Pineda-Urbina, José M. Flores-Álvarez, Juan P. Mojica-Sánchez, Jorge A. Cárdenas-Magaña, Cesar A. Villa-Martínez, Iván Delgado-Enciso

**Affiliations:** 1 Molecular Medicine Laboratory, Academic Unit of Human Medicine and Health Sciences, Autonomous University of Zacatecas, Zacatecas 98160, Mexico; marioalcalaperez@uaz.edu.mx (M.A.A.-P.); idaliagv@uaz.edu.mx (I.G.-V.); uriel.diaz.llerenas@uaz.edu.mx (U.D.-L.); margaritamf@uaz.edu.mx (M.L.M.-F.); 2 Carrera de Medicina Integral y Salud Comunitaria, Universidad para el Bienestar Benito Juárez García (UBBJ)-Sede Armería, Degollado 428, Ejido Armería, Armería 28305, Colima, Mexico; 3 Department of Molecular Medicine, School of Medicine, University of Colima, Colima 28040, Mexico; ghfuentes@ucol.mx (G.A.H.-F.); frojas@ucol.mx (F.R.-L.); 4 Faculty of Chemical Sciences, University of Colima, Colima 28400, Mexico; aaramos@ucol.mx (Á.A.R.-O.); kpineda@ucol.mx (K.P.-U.); josemanuel@ucol.mx (J.M.F.-Á.); cavm.1663@gmail.com (C.A.V.-M.); 5 State Cancerology Institute of Colima, Health Services of the Mexican Social Security Institute for Welfare (IMSS-BIENESTAR), Colima 28085, Mexico; 6 Clinical Epidemiology Research Unit, Mexican Institute of Social Security, Villa de Alvarez 28984, Colima, Mexico; jose.esquivel@imss.gob.mx; 7 Department of Engineering in Sustainable Agricultural Innovation, Tecnológico Nacional de México/Instituto Tecnológico José Mario Molina Pasquel y Henríquez, Unidad Académica Tamazula, Carretera Tamazula-Santa Rosa No. 329, Tamazula de Gordiano 49650, Jalisco, Mexico; juan.mojica@tamazula.tecmm.edu.mx; 8 División de Ciencias Exactas, Naturales y Tecnológicas, Departamento de Ciencias Computacionales e Innovación Tecnológica, Universidad de Guadalajara (CUSUR), Av. Enrique Arreola Silva No. 883, Colonia Centro, Ciudad Guzmán 49000, Jalisco, Mexico; 9 Department of Electromechanical Engineering, Tecnológico Nacional de México/Instituto Tecnológico José Mario Molina Pasquel y Henríquez, Unidad Académica Tamazula, Carretera Tamazula-Santa Rosa No. 329, Tamazula de Gordiano 49650, Jalisco, Mexico; jorge.cardenas@tamazula.tecmm.edu.mx; 10 Robert Stempel College of Public Health and Social Work, Florida International University, Miami, FL 33199, USA

**Keywords:** glyphosate, glyphosate-based herbicides, breast cancer, cancer cell proliferation, tumor growth, endocrine disruptors, breast cancer cells

## Abstract

The widespread use of glyphosate-based herbicides (GBHs) has raised concerns about their potential role in hormone-sensitive cancers such as breast cancer. This systematic review aimed to evaluate preclinical evidence on the effects of glyphosate (pure compound) or glyphosate-based herbicide formulations (GBHs) exposure on breast cancer cell proliferation and related molecular pathways. A structured search was conducted across PubMed, ScienceDirect, and Springer Nature Link, Web of Science databases, covering studies published up to 9 November 2025, following a PROSPERO-registered protocol (ID: CRD42021238350). Eligible studies included original in vitro and in vivo preclinical research using human breast cancer cell lines (e.g., MCF-7, T47D, MDA-MB-231, MCF-12A, and MCF-10A) or relevant animal models. Outcomes assessed included cell viability, proliferation, tumor growth, apoptosis, cell cycle regulation, and molecular markers associated with endocrine signaling. Two reviewers independently screened and extracted data, resolving disagreements via discussion or third-party adjudication. From an initial pool of 699 articles, seven in vitro studies met the inclusion and quality criteria. Glyphosate exposure demonstrated weak estrogenic activity in ER-positive breast cancer cells, primarily via ERα modulation and altered gene expression related to proliferation and DNA repair. GBHs showed greater cytotoxic and epigenetic effects in non-tumorigenic cells, often independent of ER signaling. No included study employed in vivo breast cancer models. Overall, preclinical evidence suggests glyphosate may act as a weak endocrine disruptor under specific conditions, but findings are limited by the short-term in vitro designs, heterogeneous methodologies, and lack of chronic or in vivo data. Further research using long-term exposure and animal models is needed to clarify potential risks and inform regulatory and public health decisions.

## 1. Introduction

Glyphosate, the active ingredient in many herbicidal formulations such as Roundup^®^, is the most widely used herbicide worldwide. In the United States, its use increased nearly 16-fold between 1992 and 2009, largely driven by the introduction of glyphosate-resistant genetically modified crops [1,2]. Given this widespread application and the potential for chronic human exposure, glyphosate has been subject to extensive toxicological evaluation. However, international health agencies have issued conflicting assessments regarding their carcinogenic potential [1,3,4,5]. Notably, in 2015, the International Agency for Research on Cancer (IARC) classified glyphosate as “probably carcinogenic to humans” (Group 2A), citing sufficient evidence of carcinogenicity in animals, limited evidence in humans—particularly for non-Hodgkin lymphoma (NHL)—and mechanistic data supporting genotoxicity and oxidative stress induction [6].

These mechanistic properties align with two of the ten hallmarks of cancer described by Hanahan in 2011 [7], which are now widely adopted in carcinogenic hazard identification by regulatory agencies such as the U.S. Environmental Protection Agency (EPA). This framework highlights whether a compound promotes carcinogenesis through mechanisms such as DNA damage, hormone receptor modulation, or disruption of cell proliferation and apoptosis [8,9].

Since the IARC’s 2015 evaluation, multiple studies have reported both epidemiological associations and mechanistic insights regarding glyphosate exposure and cancer risk. While non-Hodgkin lymphoma has been the primary focus, emerging evidence suggests that glyphosate may also influence other malignancies, including hormone-dependent cancers such as breast cancer [3,8,9,10,11,12]. In addition, recent evidence demonstrates that glyphosate can exert endocrine-disrupting activity in other hormone-responsive tissues, including prostate cells, further supporting its potential to modulate steroid-dependent cancer pathways [13]. Experimental data indicate that glyphosate can mimic estrogenic activity, promote estrogen receptor (ER) activation, and alter the expression of genes involved in cell cycle regulation and proliferation, raising concerns about its role as a potential endocrine disruptor in ER-positive breast cancer cells [14,15,16,17].

Beyond regulatory classifications, an extensive body of experimental research has explored the biological effects of glyphosate and glyphosate-based herbicides (GBHs) in mammalian systems [18]. These studies consistently describe mechanisms such as oxidative stress, mitochondrial dysfunction, endocrine disruption, altered cell cycle progression, and impaired DNA repair, all of which are relevant to carcinogenic processes. In breast tissue models specifically [19,20], GBHs have been shown to modulate estrogen receptor signaling, influence proliferation pathways such as MAPK/ERK and PI3K/AKT, and promote epigenetic modifications that may prime cells for malignant transformation [20,21,22]. Together, these mechanistic findings underscore the importance of evaluating whether glyphosate or its formulations can influence tumor-promoting or hormone-responsive pathways in breast cancer models.

In alignment with the Population, Exposure, Comparator, and Outcomes (PECO) framework [23], this systematic review was designed to identify, synthesize, and critically evaluate preclinical evidence addressing the following question: Does exposure to glyphosate or glyphosate-based herbicides, compared with unexposed controls, promote proliferation or mechanistic changes consistent with tumor progression in preclinical breast cancer models? The review focused on in vitro and in vivo experimental studies involving mammalian breast cells or tissues. Given the widespread and persistent use of glyphosate-based herbicides, clarifying their potential role in breast cancer progression through experimental evidence is essential to better understand their biological impact and possible health risks.

## 2. Materials and Methods

### 2.1. Study Design

A systematic review was conducted in accordance with the Preferred Reporting Items for Systematic Reviews and Meta-Analyses (PRISMA) checklist [24]. The objective was to improve accessibility to existing evidence by identifying, evaluating, and synthesizing findings from studies examining the effects of glyphosate exposure on the growth and proliferation of breast cancer cells in preclinical models, including both in vitro and in vivo experiments.

Because this review aims not only to identify eligible studies but also to contextualize and integrate mechanistic evidence, the methodological approach was designed to combine systematic identification of preclinical data with a narrative synthesis of biological effects. This dual structure allows the review to preserve transparency and reproducibility while providing a broader conceptual discussion of the molecular pathways influenced by glyphosate and GBHs, in line with recommendations from the reviewer regarding knowledge-based presentation.

To ensure a comprehensive evaluation of the available evidence, this review included studies using both pure glyphosate and glyphosate-based herbicide (GBH) formulations. Although GBHs contain additional surfactants and adjuvants that may independently influence toxicity, their inclusion was justified for two reasons. First, all eligible studies were required to explicitly report the concentration of glyphosate present in the formulation and to relate their findings to the corresponding dose of active ingredient, allowing comparability across experimental models. Second, preclinical literature frequently uses either pure glyphosate or commercial formulations, depending on experimental feasibility, and restricting the search to one form would have excluded relevant mechanistic data regarding breast cancer cell responses. Including both types of exposures, therefore, reflects real-world experimental diversity while enabling a more complete synthesis of biological effects attributable to the glyphosate moiety. Nonetheless, the potential contribution of formulation constituents was acknowledged during data interpretation, and results were analyzed with explicit attention to the proportion of active glyphosate reported in each study.

### 2.2. Search Strategy

A comprehensive bibliographic search was conducted from March to 9 November 2025, utilizing the electronic databases PubMed, ScienceDirect, Springer Nature Link, and Web of Science. The search targeted in vitro and preclinical animal studies assessing the effects of glyphosate on breast cancer. The search strategy included terms such as Glyphosate AND (“breast cancer” OR cancer OR tumor OR malignancy OR masses); Glyphosate AND (“in vitro studies” OR “MCF-7 cells” OR “MDA-MB-231 cells” OR “T47D cells” OR “MCF-10A cells” OR “MCF-12A cells” OR “breast epithelial cells”); and Glyphosate AND (“preclinical studies” OR “in vivo studies” OR “murine models” OR “Nu/Nu mice” OR “Balb/c mice”). The search was restricted to the title and abstract fields. The corresponding protocol for this systematic review has been registered on the PROSPERO platform (ID: 1238350).

### 2.3. Selection of Studies

No restrictions were applied regarding the year of publication, and only original research articles published in English were considered. Eligible studies examined the effects of glyphosate or glyphosate-based formulations on breast cancer using human breast cancer cell lines (MCF-7, MDA-MB-231, T47D, MCF-12A, or MCF-10A) or in vivo preclinical models, with particular attention given to studies employing immunodeficient mice (e.g., Nu/Nu or Balb/c) inoculated orthotopically or subcutaneously with breast cancer cells. To qualify, studies were required to provide original experimental data on outcomes such as cell viability and proliferation, tumor growth and progression, or the expression of molecular markers related to apoptosis or cell cycle regulation, with appropriate control groups. Highly cited or foundational studies were prioritized when relevant.

Studies were excluded if they did not directly assess the effects of glyphosate on breast cancer cells or relevant animal models, involved unrelated tumor types, or were non-original works such as reviews, editorials, letters, conference abstracts, or grey literature. Articles lacking sufficient methodological detail (e.g., missing abstracts or DOIs) or unavailable in full text, as well as duplicate records, were also excluded [25].

Two independent reviewers screened titles and abstracts, followed by full-text assessment. Discrepancies were resolved by consensus or a third reviewer. Articles were classified into three categories: (1) Included: met at least one inclusion criterion and no exclusion criteria; (2) Excluded: met at least one exclusion criterion; and (3) Uncertain: unclear cases. Any discrepancies between the two investigators were resolved by discussion or, if necessary, by consultation with a third reviewer [26,27].

### 2.4. Data Extraction

Data from included studies were systematically collected in a Microsoft Excel spreadsheet. Extracted variables included study title, type of research, experimental model, assays performed, exposure duration and/or administration route, glyphosate concentration, number of replicates, total sample size, study objectives, outcomes, measurement techniques, main findings, statistical analyses, software used, authors’ conclusions, and reported limitations [28,29]. Data extraction was performed independently by two reviewers using a standardized form. Extracted data were cross-checked for accuracy. To promote transparency and reproducibility, the complete dataset is available as Supplementary Materials accompanying this review, and it will also be deposited in the open-access repository ZENODO upon publication [30].

### 2.5. Quality Assessment of the Included Studies

The methodological quality of the included studies was evaluated using an Excel-based matrix adapted from the Quality Assessment Tool for Quantitative Studies. This framework considered four domains: study design (QCD), conduct (QCC), data analysis (QCA), and conclusions (QCCo), with each item scored using a binary system (1 = yes, 0 = no) for a maximum of 10 points per study. Studies scoring 10 points were classified as high quality, 9 points as acceptable, and 7–8 points as low quality, while those scoring below 7 points were excluded for failing to meet minimum methodological standards. Based on this assessment, five studies achieved scores of 9 or higher and were included in the final analysis [31,32].

Due to the heterogeneity of study designs and the limited number of studies, a formal certainty assessment of the evidence using approaches such as GRADE was not conducted. Nonetheless, the quality evaluation provides a systematic appraisal of the robustness and reliability of the included preclinical studies [33].

## 3. Results

### 3.1. Study Selection

**Figure 1:**
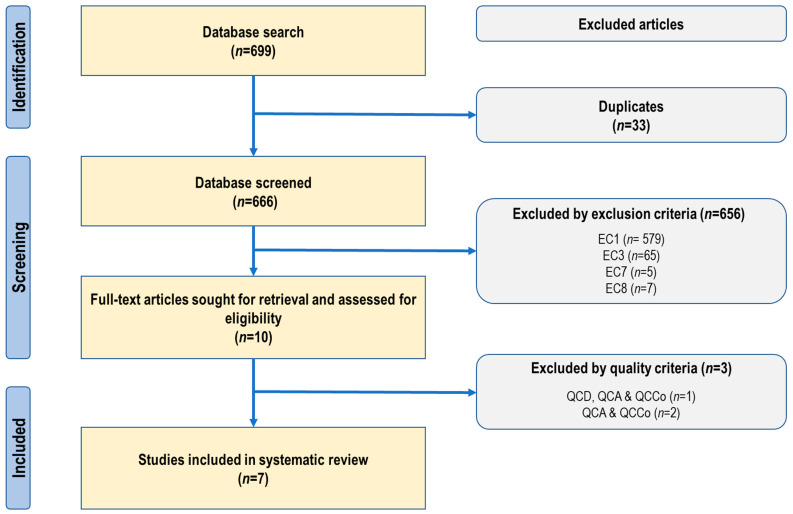
PRISMA-style flow diagram illustrates the identification, screening, and inclusion of studies for systematic review. The diagram summarizes the identification, screening, eligibility, and inclusion of studies in the systematic review evaluating the effects of glyphosate and glyphosate-based herbicides on breast cancer using in vitro and preclinical animal models. Studies were excluded based on predefined exclusion criteria (EC1, EC3, EC7, and EC8) and quality criteria: QCD, deficiencies in study design; QCA, deficiencies in data analysis; and QCCo, unsupported or inconsistent conclusions.

A total of 699 articles were identified across three databases: 257 from PubMed, 4 from Springer Nature Link, and 438 from ScienceDirect. After the removal of 33 duplicate records (24 from PubMed and 9 from ScienceDirect), 666 articles remained for screening. Screening was conducted using predefined exclusion criteria, which included EC1: Not related to the effects of glyphosate or glyphosate-based herbicides on breast cancer or involving unrelated tumor types; EC3: Non-original publications (e.g., reviews, editorials, letters, conference abstracts, or grey literature); EC7: Insufficient methodological information (e.g., missing abstract or missing DOI); and EC8: Full-text not available or inaccessible. During the title and abstract screening phase, 656 articles were excluded on the basis of these criteria. Specifically, PubMed contributed 228 exclusions (177 for EC1, 42 for EC3, 5 for EC7, and 4 for EC8), Springer Nature Link contributed 4 exclusions (1 for EC1 and 3 for EC3), and ScienceDirect contributed 424 exclusions (401 for EC1, 20 for EC3, and 3 for EC8). A total of 10 full-text articles were retrieved and evaluated—5 from PubMed and 5 from ScienceDirect. Of these, 3 PubMed articles were excluded for not meeting minimum quality requirements. One article scored 7/10 due to deficiencies in QCD, QCA, and QCCo, while two scored 8/10 due to methodological weaknesses in QCA and QCCo. Ultimately, 7 studies met both the eligibility and quality criteria and were included in the final systematic review—2 from PubMed and 5 from ScienceDirect. The full screening and selection process is summarized in Figure 1.

### 3.2. Study Characteristics

The studies included in this review were published between 2013 and 2025 and comprised exclusively in vitro experimental designs. A total of seven articles met the inclusion and quality assessment criteria: two retrieved from PubMed and five from ScienceDirect. Most of these studies utilized human breast cancer cell lines such as MCF-7, T47D, MDA-MB-231, and the non-tumorigenic MCF-12A and MCF-10A.

The primary assays employed included MTT, LDH release, ERE-luciferase reporter assays, RT-qPCR, Western blotting, confocal microscopy, and various cell viability and cytotoxicity measurements. Exposure times ranged from 6 to 72 h, with glyphosate (pure compound) or glyphosate-based herbicide formulations (GBHs) concentrations varying from 10^−12^ to 10^−3^ M. Most assays were conducted in triplicate to sextuplicate, and sample sizes fell within comparable experimental ranges. None of the studies are used in vivo animal models involving breast cancer cell inoculation, such as MCF-7 or MDA-MB-231 xenografts in Nu/Nu or Balb/c mice.

To facilitate methodological transparency and address reviewer concerns, the included studies were categorized according to the type of exposure: (a) studies using pure glyphosate as the active compound [16,19,21,34,35] and (b) studies evaluating glyphosate-based herbicide (GBH) formulations containing co-formulants such as surfactants and adjuvants [36,37]. This distinction is maintained throughout the Section 3 and Section 4 to account for the differing chemical compositions and toxicological profiles of GBHs compared with glyphosate alone.

The included studies investigated the impact of glyphosate (pure compound) and glyphosate-based herbicide formulation (GBH) exposure on cell proliferation, estrogen receptor (ER) activation, gene expression, and cellular mechanisms associated with tumorigenesis. Evaluated outcomes included cell viability, ERα phosphorylation, expression of genes linked to cell cycle regulation and DNA repair, and pathway enrichment analysis. Measurement methods comprised spectrophotometry, fluorescence assays, and luciferase-based reporting systems. One study evaluating pure glyphosate also included a comparison arm with a commercial GBH formulation, but results were analyzed independently according to exposure type.

Across these two exposure categories, pure glyphosate studies predominantly evaluated endocrine-related proliferative responses, while GBH studies more frequently reported cytotoxic, epigenetic, and DNA-repair alterations, supporting the need to interpret both evidence streams separately.

Key findings across studies suggested that glyphosate may act as a weak estrogen mimic, promoting ER-dependent signaling and cellular proliferation—particularly in ER-positive breast cancer cells. Some studies also identified effects on metabolic and repair pathways, suggesting potential endocrine-disrupting properties. Differential responses were observed between ER-positive and ER-negative lines.

Across the included studies, statistical analyses were predominantly performed using Student’s *t*-test and one-way ANOVA. The reviewed articles reported the use of statistical software such as GraphPad Prism, Partek Genomics Suite, and Omics Explorer for data analysis (software versions were reported heterogeneously across studies and are therefore not consistently available). Limitations of the reviewed literature included the exclusive reliance on short-duration in vitro assays, the absence of chronic exposure models, and the lack of in vivo confirmation, restricting extrapolation to complex biological systems.

All included studies focused on breast cancer cells, and collectively their findings suggest that under certain exposure conditions, glyphosate (pure compound) or glyphosate-based herbicide formulations (GBHs) may contribute to tumor-promoting mechanisms in hormone-responsive cellular environments. Table 1 summarizes the key study characteristics, experimental designs, and findings. The complete table can be found in Supplementary Information.

### 3.3. Risk-of-Bias Assessment

According to the customized quality assessment tool developed for this Systematic Literature Review (SLR), the included studies scored between 7 and 10 points, reflecting a range from moderate to high quality. Four studies achieved a maximum score of 10, demonstrating robust methodological rigor and low risk of bias [38]. These studies met all the criteria across the four domains, including clear objectives and hypotheses, well-described data collection methods, appropriate statistical analysis, and well-supported conclusions contextualized within existing literature. Two articles scored 9, indicating minor methodological weaknesses but sufficient quality to be included in the final synthesis. Three studies, scoring between 7 and 8, were excluded due to not meeting the threshold of ≥9 points required for the final stage.

The primary limitations identified in the excluded studies (*n* = 3) were deficiencies in the Design (QCD), Analysis (QCA), and Conclusions (QCCo) domains. Specifically, two studies failed to report confidence intervals or *p*-values to support their statistical interpretations, while all three studies offered minimal discussion of their findings in the context of previous literature. In contrast, the included studies consistently demonstrated higher methodological rigor, providing appropriate statistical analyses, referencing the software used, and clearly detailing their experimental approaches. However, none of the studies included in vivo models such as Nu/Nu or Balb/C mice, which limit the extrapolation of results to complex physiological systems. These findings highlight the importance of future research incorporating in vivo experimentation and more comprehensive statistical reporting to improve the robustness and applicability of results.

## 4. Discussion

This systematic review synthesizes experimental in vitro evidence on the effects of glyphosate (pure compound) and GBHs on breast cancer models, integrating data from seven eligible studies using human breast cancer cell lines (MCF-7, T47D, and MDA-MB-231) and non-tumorigenic breast epithelial cells (MCF-12A and MCF-10A). No study employed in vivo models such as Nu/Nu or Balb/c mice, limiting the extrapolation of cellular responses to organism-level or clinical contexts. Importantly, the objective of this review was not to infer human health risk, but to systematically integrate mechanistic signals observed across heterogeneous preclinical models in order to identify convergent biological pathways and methodological gaps.

Beyond the in vitro findings compiled here, extensive research outlines several mechanisms through which glyphosate and GBHs may influence carcinogenesis. These include oxidative stress induction, reactive oxygen species generation, DNA damage, and endocrine disruption, particularly estrogen receptor activation, aromatase modulation, and alterations in genes governing cell cycle control [36,39,40,41,42,43,44,45]. Such mechanisms provide biological plausibility for the proliferative and transcriptional alterations reported in the included studies and help contextualize their relevance to breast cancer biology [46,47].

Across the reviewed articles, glyphosate consistently displayed estrogenic activity in ER-positive cell lines. Mesnage et al. (2017) [16], Thongprakaisang et al. (2013) [19], and Muñoz et al. (2023) [21] demonstrated ERα activation, phosphorylation, degradation, and enhanced transcriptional activity, accompanied by increased proliferation at concentrations ranging from 10^−6^ to 10^−3^ M. ER-negative MDA-MB-231 cells did not exhibit proliferative responses under comparable exposure conditions, underscoring the receptor-dependent endocrine-disrupting properties of glyphosate [16,19,21]. The studies from Panis et al. (2025) [34] and Neves Rebello et al. (2025) [36] expand the molecular landscape associated with glyphosate exposure. High-throughput transcriptomic approaches identified deregulation of genes related to oxidative stress, apoptosis, and chromatin remodeling at environmentally relevant concentrations. These findings suggest that chronic low-dose GBH exposure may predispose both tumorigenic and non-tumorigenic cells to malignant transformation through persistent DNA damage and epigenetic remodeling, regardless of ER status. This broader perturbation profile emphasizes the need for integrated toxicogenomic approaches in future preclinical models. While estrogen receptor activation is a recurring finding, the reviewed evidence indicates that glyphosate- and GBH-induced effects are not limited to classical estrogen agonism, but also involve oxidative stress responses, DNA damage signaling, and epigenetic and transcriptional deregulation, including in ER-negative models [13].

Despite these insights, heterogeneity across studies, including differences in glyphosate or GBH concentrations, exposure durations, and experimental methodologies, limits comparability and hinders precise interpretation. The exclusive reliance on in vitro assays further highlights the need for in vivo models to validate mechanistic pathways and assess systemic interactions within the tumor microenvironment [48,49,50].

An additional methodological consideration concerns the inclusion of studies evaluating either pure glyphosate or glyphosate-based herbicide (GBH) formulations. Although these exposure types are not chemically identical due to the presence of surfactants and adjuvants, their combined synthesis was feasible because the eligible GBH studies explicitly reported the concentration of glyphosate corresponding to the active ingredient within each formulation. This allowed biological responses to be interpreted relative to the glyphosate moiety, facilitating comparability with studies using the pure compound. Furthermore, both exposure forms are relevant within preclinical toxicology, as pure glyphosate enables mechanistic isolation, while GBHs reflect real-world exposure conditions. Integrating both types of evidence, therefore, provides a broader characterization of potential endocrine-disrupting and cytotoxic effects, while acknowledging that co-formulants may enhance or modulate these responses [51,52]. In this context, it is important to consider that surfactants and adjuvants present in GBHs may alter cellular permeability, oxidative balance, or endocrine signaling independently of the active ingredient. Because the reviewed studies rarely isolate or quantify the effects of individual formulation components, future preclinical research should systematically evaluate coformulants to determine their mechanistic roles and to better differentiate glyphosate-driven effects from formulation-derived toxicity.

The principal strength of this SLR is its focused evaluation of glyphosate (pure compound) or GBHs in breast cancer cells using standardized assays such as MTT and RT-qPCR, enabling a consistent synthesis of cytotoxic and estrogenic outcomes. Moreover, the inclusion of studies that met predefined quality assessment criteria reinforces the reliability of the evidence presented [42,53]. Notably, four studies (Mesnage et al. (2017) [16], Thongprakaisang et al. (2013) [19], Muñoz et al. (2023) [21], and Stur et al. (2019) [37]) achieved full quality scores, as they exclusively evaluated glyphosate or GBHs and implemented clear, reproducible, and mechanistically consistent experimental designs [16,19,20,36,54]. By organizing fragmented in vitro evidence into a coherent mechanistic framework, this review helps delineate which biological endpoints are consistently affected, which remain inconclusive, and where experimental standardization is most urgently needed.

However, the absence of in vivo models and long-term exposure assessments represents a significant limitation. Without animal data, it is difficult to infer systemic responses, chronic effects, or tumorigenic potential [55,56]. Methodological variability in glyphosate or GBH concentrations and exposure times further hampers the integration of findings. Although omics-based technologies could provide deeper mechanistic resolution, their limited use in the reviewed studies restricts exploration of pathways potentially affected by glyphosate [57,58,59].

Another important limitation is the insufficient evaluation of co-formulants present in commercial GBHs. These adjuvants may enhance or modify the toxicity of glyphosate; however, they were rarely addressed despite evidence of additive or synergistic interactions [35]. Additionally, many in vitro studies employed concentrations higher than those detected in environmental or human biomonitoring data, reducing their real-world relevance. Future studies should incorporate environmentally realistic doses and consider co-formulant contributions, ideally integrating pharmacokinetics and exposure science to strengthen human health risk assessments [57,60,61,62,63].

Although glyphosate and glyphosate-based herbicides have been suggested to influence multiple hormone-responsive tumors, including ovarian, thyroid, and prostate cancers, the present review intentionally focused on breast cancer models. This decision reflects both the biological specificity of breast cancer as an estrogen-dependent malignancy and the methodological coherence required for systematic synthesis. The available preclinical studies differ substantially across hormone-related cancers in terms of exposure paradigms, cellular models, mechanistic endpoints, and outcome reporting, making cross-cancer comparisons methodologically unreliable within a single analytical framework. Concentrating on breast cancer—where in vitro models such as MCF-7, T47D, MDA-MB-231, MCF-10A, and MCF-12A are well-characterized and widely used—allowed for a more rigorous evaluation of mechanistic pathways, including estrogen receptor signaling, DNA damage responses, and metabolic alterations. Expanding this review to other tumor types would broaden the scope beyond its original objective and dilute the depth of mechanistic interpretation attainable for breast cancer specifically. Future systematic reviews could address glyphosate’s effects across multiple hormone-dependent cancers using harmonized criteria tailored to each tumor type [15,36,37]. This review further advances the field by providing a critical mechanistic synthesis rather than a descriptive summary, integrating proliferative, endocrine, genotoxic, and epigenetic responses into a coherent biological framework.

While standard cytotoxicity and estrogenic assays have been commonly used, complementary analytical technologies—such as metabolomics, proteomics, high-content imaging, and flow cytometry—could substantially enhance mechanistic insights by enabling the precise quantification of glyphosate uptake, intracellular dynamics, and downstream effects [64]. Incorporating these techniques alongside co-formulant analyses would improve mechanistic resolution and better support risk assessment frameworks [2,65,66,67,68,69,70,71].

Finally, the ecological fallacy inherent in extrapolating in vitro outcomes to human health must be acknowledged. Variability in cell line properties and experimental design complicates generalizability, underscoring the need for cautious interpretation until in vivo data are available [68]. Future research should address these knowledge gaps by implementing animal models, standardizing experimental conditions, and evaluating chronic low-dose exposures. Such efforts are essential for elucidating the full spectrum of glyphosate’s biological effects and informing regulatory and public health decisions.

Taken together, the mechanistic evidence across the included studies indicates that glyphosate and GBH exposure can converge on three major cellular axes: (i) estrogen receptor signaling and proliferation; (ii) oxidative stress and impaired DNA repair; and (iii) epigenetic and transcriptional remodeling. Although these pathways plausibly interact to promote malignant phenotypes, current evidence remains insufficient to define a unified mechanism of action. More integration, dose-relevant, and cross-platform studies are needed to resolve these mechanistic intersections.

**Figure 2:**
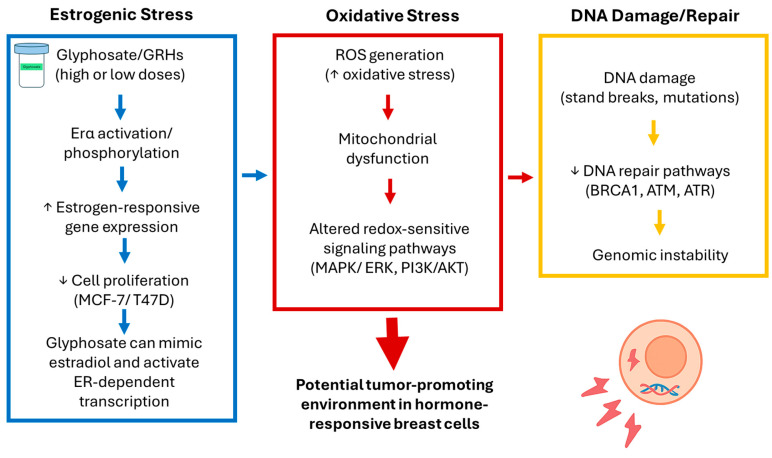
Graphical summary of the proposed mechanistic interactions triggered by glyphosate and glyphosate-based herbicides (GBHs) in breast cancer cells. Glyphosate may activate estrogen receptor α (ERα), enhance estrogen-responsive gene transcription, and promote cell proliferation in hormone-dependent breast cancer models. Concurrently, GBH exposure can increase reactive oxygen species (ROS) levels, leading to oxidative stress and redox-sensitive signaling pathway activation. Elevated ROS may also induce DNA damage while impairing DNA repair mechanisms, thereby contributing to genomic instability. The crosstalk between ER activation, oxidative stress, and DNA damage suggests that these pathways may converge to create a cellular environment conducive to tumor-promoting responses. ↑ increase in the activity. ↓ decrease in the activity.

A growing body of evidence suggests that estrogen receptor activation, oxidative stress, and DNA damage are not independent mechanisms but instead form an interconnected network that may amplify carcinogenic signaling in breast cells exposed to glyphosate or GBHs [13,69]. ER activation can increase metabolic activity and mitochondrial respiration, which in turn elevates the production of reactive oxygen species (ROS). Excessive ROS contributes to oxidative stress, promoting base modifications, strand breaks, and impaired DNA repair capacity [13,70,71]. Conversely, oxidative DNA damage can further enhance ER signaling through redox-sensitive transcriptional pathways, establishing a feed-forward loop that favors proliferation and genomic instability [13,70,71]. This mechanistic interplay provides a plausible biological framework linking endocrine disruption with genotoxic processes observed across the reviewed studies and may partially explain how chronic low-dose exposures could prime breast epithelial cells for malignant transformation (Figure 2). Uncertainty regarding human health relevance does not diminish the value of systematically characterizing early biological perturbations, particularly for compounds with widespread exposure and regulatory relevance.

## 5. Conclusions

This systematic review demonstrates that glyphosate and glyphosate-based herbicides exert measurable estrogenic and cytotoxic effects on breast cancer cell lines, particularly in hormone-responsive models. The evidence consistently shows activation of estrogen receptor pathways, disruption of genes involved in cell cycle regulation and DNA repair, and alterations in cell viability and proliferation under in vitro conditions.

Despite these findings, their relevance to human health remains uncertain. All available data derives from short-term cellular models, with no in vivo or long-term exposure studies to support broader toxicological or carcinogenic interpretations. Variability in cell lines, experimental conditions, and formulation types further underscores the need for cautious extrapolation.

Overall, the current body of evidence highlights biologically plausible mechanisms through which glyphosate may influence breast cancer-related pathways, but it is insufficient to draw definitive conclusions regarding real-world risk. Future research should prioritize standardized protocols, animal model validation, and chronic exposure assessments, as well as mechanistic analyses incorporating endocrine and epigenetic endpoints. Such efforts are essential to clarify the potential implications of glyphosate exposure for breast cancer and to support informed regulatory decision-making.

## Figures and Tables

**Table 1:** Summary of the experimental characteristics and main outcomes of studies evaluating glyphosate or glyphosate-based herbicides (GBHs) in human breast cell models.

Item	Type of Study	Experimental Model	Glyphosate Concentration	Aim of the Study	Main Findings
1a	in vitro	Human breast cell lines: MCF-7 (ER^+^ tumorigenic) and MCF-12A (non-tumorigenic)	50–500 ppb	Evaluate the toxicological effects of glyphosate (and other pesticides) at environmentally relevant concentrations on a cancerous vs. a non-cancerous breast cell line, assessing multiple cellular endpoints	Glyphosate reduced viability, ATP levels, and ROS in breast cells, triggered apoptosis, and showed endocrine-disrupting effects by altering hormone receptor expression and estradiol secretion [34].
2a	in vitro	Human breast cancer cells (MCF-7, T47D, MDA-MB-231)	1 × 10^−8^–1 × 10^−3^ M	Evaluate estrogenic effects in ER^+^ and ER^–^ cells	Glyphosate induces weak ERα-mediated proliferation and gene expression [21]
3a	in vitro	T47D-KBluc, MCF-7, MDA-MB-231	10^−12^–10^−6^ M	Assess ER-mediated transcription and proliferation	Glyphosate acts as weak ER agonist; GBHs less effective [16]
4a	in vitro	T47D, T47D-KBluc, MDA-MB-231	10^−12^–10^−6^ M	Investigate ER-mediated effects and interaction with genistein	Low-dose glyphosate shows estrogenic effects; genistein enhances them [19]
5a	in vitro	MCF-7 (tumorigenic) and MCF-12A (non-tumorigenic)	230 pM–2.3 µM	Compare toxicological effects of pesticides at child-relevant concentrations	Glyphosate alters energy metabolism and shows endocrine-disrupting effects in both models [35]
6b	in vitro	Non-tumorigenic human breast epithelial cells (MCF10A) and tumorigenic breast cancer cells (MCF7, ER^+^; MDA-MB-231, ER^−^)	GBHs at 0.000001–1% (*v*/*v*) (≈0.0048 µg/mL to 4.8 mg/mL glyphosate; ~0.03 µM–28 mM)	Verify the impact of a glyphosate-based herbicide on non-tumor and tumor breast cells by analyzing breast cancer gene expression and potential epigenetic changes linked to cancer risk	GBHs showed greater toxicity in non-tumor MCF10A cells than in cancer cells, reducing viability and suppressing BRCA genes. It altered DNA repair gene expression without estrogenic activity and induced complex, non-reversible epigenetic effects [36].
7b	in vitro	MCF-7 (ER^+^) and MDA-MB-468 (ER^–^)	0.01–0.30% *v/v* (≈1.1 mM)	Identify gene expression and pathway changes after short-term exposure	Low-dose GBHs and AMPA affect cell cycle and DNA repair independently of ER status [37]

Concentrations are reported as originally stated by the authors, including ppb, percent volume/volume (*v*/*v*), and molarity (M). ER^+^ and ER^−^ indicate estrogen receptor-positive and -negative cell lines, respectively. Reported effects include cytotoxicity, endocrine-disrupting activity, gene expression changes, and epigenetic alterations as described in the original publications. a Studies using glyphosate as a pure compound. b Studies using glyphosate-based herbicide formulations (GBHs).

## Data Availability

No new data were created or analyzed in this study. Data sharing is not applicable to this article.

## Supplementary Materials

The following supporting information can be downloaded at: https://www.mdpi.com/article/10.3390/toxics14010026/s1, Table S1. Complete description of study characteristics.

## Abbreviations

The following abbreviations are used in this manuscript:

GBHs	Glyphosate-Based Herbicide Formulations
PROSPERO	Prospective Register Of Systematic Reviews Only
SRL	Systematic Literature Review
IARC	International Agency for Research on Cancer
EPA	U.S. Environmental Protection Agency
PECO	Population, Exposure, Comparator, and Outcomes
PRISMA	Preferred Reporting Items for Systematic Reviews and Meta-Analyses
DOI	Digital Object Identifier
IC	Inclusion Criteria
EC	Exclusion Criteria
QCD	Quality Criteria Design
QCC	Quality Criteria Conduct
QCA	Quality Criteria Analysis
QCCo	Quality Criteria of Conclusion
MTT	Metil Thiazolyl Tetrazolium
LDH	Lactate Dehydrogenase
ERE	Estrogen Response Element
RT-qPCR	Reverse Transcription quantitative Polymerase Chain Reaction
ER	Estrogen Receptor
DNA	Deoxyribonucleic Acid
ANOVA	Analysis of Variance
