# Supplementary material for: Glyphosate as an Emerging Environmental Pollutant and Its Effects on Breast Cancer Cell Proliferation: A Systematic Literature Review of Preclinical Evidence
Source: Toxics. 2025 Dec 26;14(1):26. doi: 10.3390/toxics14010026 (PMC12846237; doi:10.3390/toxics14010026)
Supplement: Supplementary file 1 [file toxics-14-00026-s001.zip › toxics-4035200-supplementary.pdf]

Table S1. Complete description of study characteristics

Item	Type of study	Experimental model	Tests performed	Exposure time / route of administration	Glyphosate concentration	Aim of the study	Author's conclusion	Limitations
**1**	*In vitro* experimental study	Human breast cancer cell lines (MCF-7, T47D, MDA-MB-231)	MTT assay, LDH release, ERE-luciferase assay, RT-qPCR, Western blot, ICC, confocal microscopy	24–48 h; in vitro via culture medium	1×10^{-8} to 1×10^{-3} M	Evaluate estrogenic effects of glyphosate in ER-positive and ER-negative breast cancer cells	Glyphosate induces ERα-mediated proliferation and gene expression; weak estrogenic activity	Only in vitro evidence; no in vivo confirmation
**2**	*In vitro* experimental study	Human breast cancer cell lines: MCF-7 (ER+) and MDA-MB-468 (ER–)	MTT assay, transcriptome profiling (HTA 2.0 microarray), PCA, RMA, pathway enrichment analysis	Viability: 3, 15, 24, 48 h; Transcriptome: 48 h	Roundup® 0.01–0.30% v/v (viability); 0.05% v/v ≈ 1.1 mM glyphosate (transcriptomic)	Identify gene expression and pathway changes after low-dose, short-term Roundup and AMPA exposure in ER+ and ER– cells	Roundup and AMPA caused cellular damage at low doses, primarily affecting cell cycle and DNA repair, independent of ER status	Short-term exposure; in vitro only; limited extrapolation to in vivo
**3**	*in vitro* comparative study	T47D-KBluc (luciferase reporter), MCF-7, MDA-MB-231	ERE-luciferase reporter assay, E-screen assay	24–48 h; in vitro with culture medium	10^{-12} to 10^{-6} M	Assess glyphosate and GBHs on ER-mediated transcription and proliferation	Glyphosate acts as a weak ER agonist under specific conditions; GBHs less effective	Possible residual estrogen in media; variability in ERα expression among cell lines
**4**	*In vitro* mechanistic study	T47D, T47D-KBluc, MDA-MB-231	MTT assay, luciferase assay, Western blot, cell counting	6–24 h; in vitro in hormone-deprivation media	10^{-12} to 10^{-6} M	Investigate ER-mediated effects of glyphosate and interaction with genistein	Low-dose glyphosate shows estrogenic effects; genistein enhances these effects	No animal models; combined effects need in vivo validation
**5**	In vitro comparative experimental study	Human mammary cell lines: MCF-7 (tumorigenic) and MCF-12A (non-tumorigenic)	MTS viability, CyQuant proliferation, Annexin V RealTime-Glo (apoptosis/necrosis), Mitochondrial ToxGlo (ATP), ROS assay, 17β-estradiol ELISA, RT-qPCR (nuclear receptor expression)	Viability/Proliferation/ATP/ELISA/qPCR: 72 h; ROS: 24 h; Apoptosis/necrosis: 0–8 h (measured at 24 h)	230 pM – 2.3 µM (5 serial dilutions)	Compare toxicological effects of CPF, IMI, and GLY at child-relevant concentrations in two mammary cell models	Glyphosate altered energy metabolism and showed endocrine-disrupting effects at human-relevant concentrations	In vitro only; lack of systemic cofactors; limited exposure times and concentrations
6	Glyphosate-based herbicides at low doses affect canonical pathways in estrogen positive and negative breast cancer cell lines	Experimental *in vitro* study (toxicogenomic analysis in cultured breast cancer cells)	ER-positive (MCF-7) and ER-negative (MDA-MB-468) human breast cancer cell lines	Cell viability assay (MTT) and genome-wide gene expression profiling (transcriptomics) after pesticide exposure; pathway enrichment analysis of deregulated genes	48 h exposure in vitro (glyphosate-based formulation or metabolite added to culture medium)	Roundup Original formulation at 0.05% v/v (≈1.1 mM glyphosate); comparison with AMPA at 10 mM	Technical triplicates (MTT assays in triplicate wells)	Biological triplicates (n = 3 independent sample replicates for microarray)
7	Comparison of the Toxicological Effects of Pesticides in Non-Tumorigenic MCF-12A and Tumorigenic MCF-7 Human Breast Cells	Experimental *in vitro* study (toxicological assays on cultured human breast cells)	Human breast cell lines: MCF-7 (ER^+ tumorigenic) and MCF-12A (non-tumorigenic)	Cytotoxicity (cell viability), cell proliferation, apoptosis and necrosis, intracellular ROS, ATP levels, 17β-estradiol secretion, and gene expression of nuclear receptors	72 h exposure in culture (cells treated in medium)	Five concentrations from 230 pM to 2.3 µM (10^{-7}–10^{-6} M; 10-fold serial dilutions)	Triplicate wells per condition	3 independent experiments (n = 3)
